# Preschool Teachers’ Emotional Competence and Teacher Self-Efficacy towards Preschool Performance in Zhejiang Province of China

**DOI:** 10.3390/bs14040280

**Published:** 2024-03-28

**Authors:** Xiaolu Ye, Nor Aniza Ahmad, Nur Aimi Nasuha Burhanuddin, Meng Na, Danwei Li

**Affiliations:** 1 Department of Foundations of Education, Faculty of Educational Studies, Universiti Putra Malaysia (UPM), Serdang 43400, Selangor Darul Ehsan, Malaysia; gs61759@student.upm.edu.my (X.Y.); aiminasuha@upm.edu.my (N.A.N.B.); zadsxy@163.com (D.L.); 2 Graduate School of Business, Universiti Kebangsaan Malaysia (UKM), Bangi 43600, Selangor, Malaysia; zp05840@siswa.ukm.edu.my

**Keywords:** emotional competencies, preschool performance, teacher’s self-efficacy, teaching experience

## Abstract

This study investigates the interplay between emotional competence, self-efficacy, and teaching experience in determining preschool teachers’ performance. Drawing on Bandura’s Theory of Self-Efficacy, Goleman’s Emotional Intelligence Theory, and Bronfenbrenner’s Ecological Systems Theory, the research employs a quantitative approach, analyzing responses from preschool teachers in Zhejiang province, China. Key findings reveal that emotional competence significantly predicts teachers’ performance and self-efficacy, with self-efficacy further mediating this relationship. Interestingly, while teaching experience moderates the impact of emotional competence on self-efficacy, it does not significantly influence the relationship between self-efficacy and teaching performance. The study underscores the critical role of emotional competence in teaching efficacy and highlights the complexity of how teaching experience interacts with these dynamics. These insights are crucial for developing targeted interventions in teacher training programs, emphasizing emotional skills and self-belief as key drivers of effective teaching in early-childhood education.

## 1. Introduction

The realm of early-childhood education, critical for laying the foundation in children’s formative years, faces a plethora of challenges that substantially affect the performance and well-being of preschool teachers, particularly in regions with unique socio-cultural and educational landscapes like Zhejiang province [1]. This province, known for its rapid economic development, vibrant cultural heritage, and innovative educational policies, presents a distinct context for examining the performance and well-being of preschool teachers. Despite its advancements, the sector in Zhejiang faces challenges similar to those globally—predominantly, the burnout and mental health of educators [2]. Recent surveys, including a comprehensive 2022 nationwide poll, have disclosed that almost half of preschool teachers are grappling with high levels of stress and burnout [3]. This issue has been further intensified by the COVID-19 pandemic, which has contributed to heightened levels of depression amongst these educators, stemming from increased physical, emotional, and financial strains [4].

In Zhejiang province, a region characterized by its rapid economic development and cultural wealth, the realm of early-childhood education encounters a distinctive set of challenges and opportunities [5]. Despite the province’s economic vigor, driven largely by a thriving private sector and foreign investments, there exists a pronounced disparity in the allocation of educational resources [6]. This discrepancy is most evident between urban and rural locales [7], as well as between private and state-run preschools [8], critically influencing both the well-being of educators and the overall quality of education provided [9]. Furthermore, while Zhejiang is renowned for its cultural heritage [10] and efforts to implement cutting-edge educational policies [11], such as the incorporation of technology in teaching and the enhancement of teacher training programs, these benefits are not uniformly distributed. This results in a complex socio-economic and cultural environment that significantly affects the efficacy of early-childhood education. The existing urban–rural divide, alongside the diversity in the types of preschool institutions, presents substantial challenges, including the need to adapt to continuously changing educational standards and to address disparities in resource availability [12,13]. Additionally, Zhejiang’s varied geographical and economic landscapes lead to differences in educational quality and teacher experiences across the region [14].

Additionally, Zhejiang’s early-childhood-education sector contends with financial limitations [3]. The stagnation in funding and low compensation rates for teachers adversely impact the overall quality of early-childhood education [15,16]. The average yearly salary of preschool educators remains significantly lower than their counterparts in K-12 settings [3], and there has been only a marginal rise in per-pupil expenditure over the last two decades. This fiscal predicament is exacerbated by a shortage of adequate professional development opportunities for early-childhood teachers [17], thereby curtailing their ability to stay abreast of evolving educational practices and research.

A nuanced exploration of Zhejiang’s unique context reveals the importance of understanding the interplay between self-efficacy, emotional competence, and classroom performance within its socio-cultural and educational framework [18,19,20]. Yet, a significant issue in the existing literature is that the segmented approach to understanding the interplay and collective impact of these factors on teaching effectiveness within Zhejiang is notably absent. While individual studies have explored the roles of self-efficacy (teachers’ confidence in their capability to handle classroom situations) and emotional competence (the ability to perceive, understand, regulate, and express emotions), there is a gap in integrative research examining their joint effect on pedagogical success [19,21,22].

The disparity in educational resources and the uneven distribution of innovative policies in Zhejiang province not only impacts the quality of early-childhood education but also underscores the necessity for comprehensive research. There is a pressing need to delve into the roles of self-efficacy and emotional competence among educators, understanding how these factors influence teaching effectiveness within the province’s diverse settings. Recognizing this need paves the way for the development of targeted interventions designed to enhance teacher professional development. Emphasizing the use of educators’ unique educational backgrounds and specialties is crucial in meeting the varied needs of young learners. Consequently, a thorough examination of how educator attributes interact with Zhejiang’s distinctive socio-cultural and educational context is essential. This approach aims to address the identified gaps and challenges, ultimately improving early-childhood education’s efficacy in this unique environment.

This problem is compounded by the lack of in-depth examination of the mediating and moderating roles of these factors. The extent to which self-efficacy mediates the relationship between emotional competence and teaching performance remains unclear. The complexity of Zhejiang’s educational landscape also prompts questions:

- Does a teacher’s belief in their abilities bolster the translation of emotional skills into effective teaching practices?

Moreover, the role of teaching experience as a moderating factor in this dynamic is yet to be fully understood.

- How does the duration and depth of a teacher’s classroom experience impact the influence of emotional competence and self-efficacy on their teaching effectiveness?

This ambiguity impedes the development of focused strategies and training programs aimed at enhancing teacher performance and does not adequately address the evolving challenges in early-childhood education, such as technological integration, cultural and socio-political shifts, and the management of increasingly diverse student needs.

The research gap identified is the absence of a comprehensive analysis of the relationships between self-efficacy, emotional competence, and classroom performance [17,18]. Furthermore, the potential roles of self-efficacy as a mediator in the relationship between emotional competence and teaching performance, and of teaching experience as a moderator in these relationships, are yet to be thoroughly explored [21,23]. This gap poses a significant hurdle in devising effective strategies for augmenting preschool teachers’ professional development and instructional practices.

This study aims to dissect the complex interplay between emotional competence, self-efficacy, and pedagogical effectiveness within the unique socio-cultural and educational context of Zhejiang province. The novelty of this study lies in its methodological and theoretical integration, bringing a unique perspective to the exploration of early-childhood education dynamics. Firstly, the utilization of Partial Least Squares Structural Equation Modeling (PLS-SEM) alongside Necessary Condition Analysis (NCA) testing represents an innovative approach in educational research. PLS-SEM provides robust modeling of complex relationships between variables, while NCA offers a rigorous examination of essential conditions required for desired outcomes. This dual-method approach ensures a comprehensive analysis of the intricate relationships among key factors influencing preschool teachers’ performance. Secondly, the theoretical underpinning of this study integrates Bandura’s Theory of Self-Efficacy [24], Goleman’s Emotional Intelligence Theory [25], and Bronfenbrenner’s Ecological Systems Theory [26], creating a multidimensional framework. This integration allows for a nuanced understanding of how personal psychological attributes (self-efficacy and emotional intelligence) and broader environmental factors (as outlined in Bronfenbrenner’s theory) collectively impact the effectiveness of preschool educators. Such a synthesis of diverse theories and advanced methodological approaches positions this study at the forefront of educational research, potentially offering groundbreaking insights into teacher performance in early-childhood education.

The anticipated findings from this study are expected to offer valuable insights into the determinants influencing the quality of early-childhood education in Zhejiang province. By elucidating these dynamics, the research seeks to inform and influence policy and practice, with the goal of enhancing the professional development of preschool educators and, consequently, enriching the educational experiences of young children. This research is poised to contribute significantly to the academic discourse in the field of early-childhood education and provide practical guidance for educators, administrators, and policymakers both within China and globally.

## 2. Emotional Competence and Self-Efficacy

Early-childhood education, serving as a foundational pillar for children’s growth and development, is significantly influenced by the quality of preschool programs. Ellen and Barnett [27] advocate for a focus on enhancing the quality of these programs, recognizing their critical role in children’s early development. The Governor’s Cabinet on Children and Families further emphasizes the importance of studies aimed at improving preschool programs, highlighting the need to explore teacher effectiveness in fostering crucial social skills and confidence in children.

Central to the discussion is the concept of teacher efficacy, as expounded by Hughes et al. [28], which significantly influences the learning process of children. Effective teachers, as Gibbs [29] notes, exhibit increased confidence in their methodologies, thereby positively impacting children’s achievement and development. Sutton and Wheatley [30] delve deeper into the emotional aspects of teaching, observing that teachers’ self-efficacy levels are closely intertwined with their emotional states, underlining the complex relationship between emotions and teaching effectiveness.

The role of teachers extends beyond mere instruction to encompass emotional competence, a skill crucial for managing personal emotions and understanding others’ emotions, especially in the context of preschool education [31,32]. Positive emotional states, as Mujis and Harris [33] argue, contribute to a more harmonious and supportive educational environment.

However, teachers today face challenges in connecting with diverse student personalities, further complicated by evolving responsibilities, expectations, and educational trends [34]. This dynamic landscape underscores the necessity for continuous development in preschool education, emphasizing the need for quality teachers who can inspire and motivate students.

Van Der Sea and Schakel [35] discuss the challenges in creating effective learning environments, a task made increasingly complex by various disciplinary issues emerging in the post-modern era. Teacher effectiveness is closely associated with their self-efficacy [36], a factor acknowledged by the Department of Education and Training as crucial for student achievement and overall school effectiveness. Yet, understanding the elements that contribute to enhancing teachers’ self-efficacy remains an open question [37,38]. The scarcity of empirical studies focusing on non-observable variables like emotions complicates discussions on quality improvement in education [39]. Sutton and Wheatley [30] suggest that the enhancement of teacher efficacy may be linked to teachers’ emotional states, necessitating further research into the impact of emotions on teacher efficacy.

The multifaceted nature of preschool education, highlights the integral roles of emotional competence and self-efficacy in teacher effectiveness. It underscores the need for further research to deepen our understanding of how these factors interact and influence the quality of early-childhood education.

## 3. Preschool Teachers Performance

Recent research in early-childhood education underscores a myriad of factors that influence the performance of preschool teachers. Creativity and organizational skills, as highlighted by researchers such as Dörterler [40], Güven [41], Titrek [42], and Yildiz [43], are essential for crafting engaging and stimulating learning environments tailored to the diverse needs of young children. Additionally, the capacity for critical reflection on teaching practices, a crucial aspect of teacher performance, enables continual enhancement of educational strategies. This reflective approach, coupled with an innate desire for self-development and creative growth as emphasized by Khanova [44] and Khanova [44], is indispensable in the evolving landscape of early-childhood education.

The significance of a teacher’s passion for the profession, identified by Kim [45] as a key influencer of beliefs and efficacy, is apparent in its profound impact on teaching methodologies and student learning experiences. Educational qualifications and specialized training also play a pivotal role, with studies by Whitebook et al. [46] showing a direct correlation with the quality of preschool education, thereby underscoring the importance of ongoing professional development.

While quality teaching in preschools positively influences child development, school readiness, and long-term educational outcomes, teachers encounter challenges such as addressing the diverse needs of students, managing work-related stress, engaging parents effectively, and adapting to changes in technology and curriculum. Tackling these challenges is essential for augmenting the quality of early-childhood education and, by extension, the performance of preschool teachers. A comprehensive understanding of these elements is critical for shaping policy and practice in early-childhood education, ensuring that educators are fully equipped to nurture and guide the development and success of young learners.

## 4. Theoretical Understanding

In this study, we integrate Bandura’s Theory of Self-Efficacy [47], Goleman’s Emotional Intelligence Theory [25], and Bronfenbrenner’s Ecological Systems Theory [26] to construct a comprehensive theoretical framework. This approach aims to examine the multifaceted factors influencing preschool teacher performance, blending individual psychological aspects with broader socio-environmental contexts.

Bandura’s concept of self-efficacy, suggesting that an individual’s belief in their ability to succeed in specific situations significantly shapes their approach to tasks [47], is central to our analysis. This framework is applied to assess how preschool teachers’ self-efficacy beliefs in Zhejiang province influence their teaching methods, particularly focusing on motivation and resilience in diverse educational settings. Bandura’s theory is crucial in understanding the relationship between preschool teachers’ emotional competence and their performance. Studies indicate that pre-service teachers with higher self-efficacy are more likely to continue in their careers [48], and emotional intelligence and self-esteem are key predictors of teacher self-efficacy [49]. Factors influencing the development of self-efficacy during student teaching include teachers’ psychosocial characteristics, which affect classroom quality and attitudes towards challenging students [50]. The multi-faceted nature of teacher efficacy, encompassing personal, outcome, and teaching efficacy, has been explored [51]. Furthermore, preschool teachers’ self-efficacy beliefs positively correlate with their teaching attitudes [52], and completing a degree can enhance practicing teachers’ self-efficacy [53].

Bronfenbrenner’s Ecological Systems Theory, which emphasizes the impact of environmental factors from immediate settings to broader societal contexts on individual development [26], is another pillar of our theoretical framework. Additionally, our study incorporates Goleman’s Emotional Intelligence Theory, highlighting elements like self-awareness, self-regulation, motivation, empathy, and social skills as vital for effective interpersonal interactions and self-management [25]. This theory is used to examine the role of emotional intelligence in teachers’ classroom management, student interactions, and overall professional performance. Research consistently shows a positive link between emotional intelligence and teacher efficacy [49,54], particularly pertinent in preschool education where emotional competence is crucial [55]. Emotional intelligence positively affects early-childhood teachers’ self-concept and self-efficacy, influences job performance [56], and can be developed through pre-service education [57]. However, the specific impact of emotional intelligence on preschool teachers’ emotional competence and self-efficacy requires further investigation.

Bronfenbrenner’s theory offers a valuable framework for understanding the interplay of factors influencing preschool teachers’ emotional competence and self-efficacy, and ultimately, preschool performance. Tudge [58] and Denham [59] highlight the significance of preschoolers’ engagement in school-relevant activities and teachers’ role in socializing emotional competence. Studies by Jennings [50] investigate the connection between teachers’ psychosocial traits and their ability to foster supportive learning environments. Additionally, Nissen [1] and Garner [60] stress the importance of emotional competence and teacher–child relationships in promoting developmental competence.

The theoretical foundation of this investigation is strategically crafted to dissect the intricate dynamics of preschool teacher performance within Zhejiang province, China. It synthesizes Bandura’s Theory of Self-Efficacy [47], Goleman’s Emotional Intelligence Theory [25], and Bronfenbrenner’s Ecological Systems Theory [26] into a cohesive framework. This amalgamation is pivotal for several reasons. First, Bandura’s Theory of Self-Efficacy underpins the exploration of how teachers’ convictions in their efficacy to enact meaningful learning and classroom management significantly impact their instructional effectiveness. The application of this theory is particularly salient in Zhejiang province, where educational contexts and expectations are markedly diverse. Delving into the self-efficacy of teachers offers profound insights into their motivation and resilience across varied educational landscapes [47]. Second, the incorporation of Goleman’s Emotional Intelligence Theory is vital for assessing the critical role of emotional capabilities in educational instruction. These capabilities are indispensable for navigating classroom dynamics, cultivating positive student–teacher interactions, and bolstering student learning outcomes. The cultural prioritization of harmony and communal well-being in Chinese society renders the emotional intelligence of educators especially significant in fostering conducive learning atmospheres [25]. Lastly, Bronfenbrenner’s Ecological Systems Theory provides a holistic lens through which the wider socio-environmental contexts that influence teaching experiences and outcomes can be examined. This approach is acutely relevant to the regional context of Zhejiang province, reflecting upon how local policies, communal expectations, and cultural values shape pedagogical practices and educational achievements [26].

**Figure 1:**
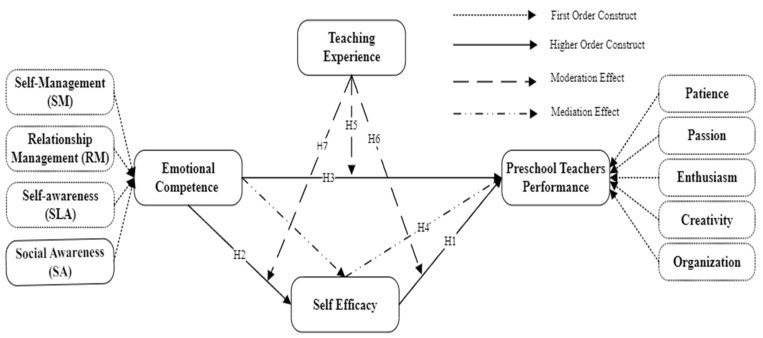
Research framework.

By intertwining these distinct yet complementary theoretical perspectives, the study seeks to offer a comprehensive overview of the elements affecting preschool teachers’ performance in Zhejiang province. This nuanced integration acknowledges the interrelation between individual competencies and broader systemic influences. The convergence of these theoretical models not only augments the scholarly discourse on early-childhood education but also provides actionable insights for the development of targeted teacher training and support mechanisms within the distinct cultural and educational milieu of China. This scholarly endeavor underscores the significance of an integrated theoretical framework in illuminating the multifaceted factors that influence preschool teacher performance, particularly within the unique context of Zhejiang province, China (refer to Figure 1). Through this comprehensive approach, the investigation aims to contribute meaningfully to both academic knowledge and practical applications in early-childhood education.

## 5. Hypothesis Development

### 5.1. Self-Efficacy and Preschool Teachers’ Performance

Research has consistently highlighted self-efficacy as a pivotal predictor of teaching performance. Studies by Cocca [61] and Guo [62] substantiate this assertion, with Guo particularly emphasizing the role of collaboration and children’s engagement. Further support is provided by Bay [63] and Şenol [64], who found a positive relationship between self-efficacy and professional skills, noting that experienced teachers exhibit higher self-efficacy. Arslan [65] and Infurna [66] extend this notion, illustrating a positive relationship between individual self-efficacy and collective self-efficacy, and identifying teaching experience and job satisfaction as significant predictors. Klassen [67] and Ortan [68] further corroborate the significant correlation between self-efficacy and teaching effectiveness, with Ortan particularly highlighting its impact on job satisfaction and well-being. Based on these findings, the first hypothesis is proposed:

**Hypothesis 1** **(H1).**  *There is a statistically significant correlation between self-efficacy and preschool teachers’ performance.*

### 5.2. Emotional Competence and Self-Efficacy

The relationship between emotional intelligence and self-efficacy has been well-documented in the literature. Azizian [69] and Yunalia [70] found a strong positive correlation between these constructs, with Azizian identifying specific components of emotional intelligence that were correlated with self-efficacy. Monica [71] and Coetzee [72] supported this correlation, linking emotional intelligence to success and a positive relationship with self-esteem. Easton [73] and Meilstrup [74] also observed a significant correlation between emotional intelligence and self-efficacy, with Meilstrup noting its potential in reducing socioeconomic disparities in emotional symptoms. Huizenga [75] proposed that emotional competencies like self-control and resilience are predictors of performance, which may also be linked to self-efficacy. Gharetepeh [76] identified emotional intelligence as a predictor of self-efficacy, especially in students with varying academic achievements. These collective studies provide robust evidence for a significant positive correlation between emotional competence and self-efficacy, leading to the second hypothesis:

**Hypothesis 2** **(H2).**  *There is a statistically significant correlation between emotional competence and self-efficacy.*

### 5.3. Emotional Competence and Preschool Teachers’ Performance 

The role of emotional competence in preschool education has been extensively explored in the literature. Garner [60] found a direct association between emotion knowledge and children’s school competence. Pakarinen [77] and Morris [78] highlighted the significance of teacher–child relationships and emotion socialization practices in fostering social and emotional competence. Denham [59] and Brock (2014) demonstrated the long-term effects of emotional competence on social behavior. The importance of teachers’ emotional support in developing children’s emotion regulation was emphasized by Denham [59] and Miller [79]. Additionally, Trentacosta [80] identified emotion regulation as a critical predictor of academic competence. These studies collectively underscore the significance of emotional competence in preschool education, forming the basis for the third hypothesis:

**Hypothesis 3** **(H3).**  *There is a statistically significant correlation between emotional competence and preschool teachers’ performance.*

### 5.4. Self-Efficacy as a Mediator

The influence of emotional competence, encompassing aspects such as emotion knowledge and emotional labor, on preschool teachers’ performance has been substantiated in studies by Garner [60] and Fu [81]. This impact is mediated through self-efficacy, a factor positively influenced by emotional intelligence and support, as noted by Valente [54], Kikas [82], and Şahin [49]. The role of teachers in providing quality emotional support is crucial in enhancing social competence among preschoolers, as evidenced by Pakarinen [77]. However, the interplay between emotional competence and self-efficacy in affecting teachers’ performance is intricate, influenced by variables including teacher collaboration and children’s engagement [62].

**Hypothesis 4** **(H4).**  *Self-efficacy mediates the influence of emotional competence on preschool teachers’ performance.*

### 5.5. Teaching Experience as a Moderator

The significance of emotional labor and intelligence in influencing teacher performance is highlighted in research by Brown [83] and Valente [54]. Bailey [84] emphasizes the importance of consistent emotional support in classrooms. The role of emotional competence in children’s developmental outcomes, including the impact of teachers’ emotion socialization behaviors, is further supported by studies from Garner [60], Denham [59], and Morris [78]. Pakarinen [77] and Curby [85] underscore the importance of teacher–child interactions in fostering children’s social competence. Collectively, these findings suggest a moderating role of teaching experience in the relationship between emotional competence and teacher performance.

**Hypothesis 5** **(H5).**  *Teaching experience moderates the relationship between emotional competence and preschool teachers’ performance.*

The correlation between self-efficacy and performance has been found to be positive in some studies [52,61,63] but non-significant in others [62,66]. The influence of teaching experience on this relationship remains ambiguous, with divergent findings in the literature. While some studies indicate no significant impact of teaching experience on self-efficacy [62,86], others identify it as a significant predictor [66]. This area warrants further investigation.

**Hypothesis 6** **(H6).**  *Teaching experience moderates the relationship between self-efficacy and preschool teachers’ performance.*

A consistent positive relationship between emotional competence and self-efficacy in teachers is evident in the literature [54,87,88,89,90]. This relationship is often mediated by factors such as teaching performance, work engagement, and professional development participation. However, the role of teaching experience in this dynamic is not well-defined. While some studies report no moderating effect [87,88], others suggest a potential enhancing effect [89], indicating the need for more research in this area.

**Hypothesis 7** **(H7).**  *Teaching experience moderates the relationship between emotional competence and self-efficacy.*

## 6. Research Methodology

This study is deeply embedded within the rich socio-economic and educational terrains of Zhejiang province, China, leveraging a robust quantitative methodology to scrutinize the emotional competence and self-efficacy of preschool teachers. The province’s distinct educational setting, marked by stark disparities in urbanization, resource availability, and cultural practices, required a tailored approach to data collection and analysis. To counteract potential biases from the heterogeneity of preschool environments across Zhejiang, we employed a stratified random sampling technique, ensuring a representative sample from the province’s diverse regions: northern, southern, eastern, and western. This methodological detail was crucial for enhancing the study’s external validity and the generalizability of its findings.

The sampling strategy commenced with the division of Zhejiang’s preschools into 12 strata, based on a combination of geographic location (north, south, east, west) and urbanization level (urban, semi-urban, rural), with each further divided into private and state-run categories. From the approximately 800 preschools identified, we aimed to randomly select schools from each stratum, aiming for proportional representation. For example, if aiming for a 10% sample from each stratum, we anticipated selecting around 80 schools in total, ensuring a mix that reflects the actual distribution of preschool types across the province.

For our final sample size determination, we utilized G*Power 3, targeting 580 respondents to achieve a power of 0.95, which significantly exceeds the standard requirements for statistical analyses in social science research. This choice was driven by the need for a large and diverse sample to adequately represent the province’s preschool teacher population, considering the complex interplay of factors influencing their professional well-being and effectiveness.

Prior to launching the survey, a comprehensive pre-testing phase was conducted with approximately 30 preschool teachers and a panel of subject matter experts. This phase served to refine the survey instrument, ensuring questions were clear, culturally appropriate, and resonant with the target demographic. The survey underwent a meticulous translation process by bilingual education experts, followed by a back-translation to verify the accuracy and integrity of the translation. This step was critical for ensuring the questionnaire was linguistically and conceptually aligned with the participants’ contexts.

In the sampling process, we randomly selected teachers from the chosen schools, ensuring each geographic and institutional stratum was adequately represented. For instance, from each selected school, around 7 to 10 teachers were randomly chosen, depending on the school’s size and teacher population, to meet our target respondent count while maintaining the diversity of our sample.

Ethical considerations were at the forefront of our methodology. All participants were informed about the study’s objectives, their role, the confidentiality of their responses, and their right to withdraw at any time. Informed consent was obtained from every participant, with the study’s protocol receiving approval from an institutional review board to ensure compliance with ethical standards.

This detailed methodology, from the stratified sampling design to the ethical safeguards and rigorous pre-testing of the survey tool, allows this study to accurately explore the nuanced realities of preschool teachers in Zhejiang. By investigating the interplay between emotional competence and self-efficacy within this unique educational context, the research aims to provide insights that could inform policy and practice, enhancing early-childhood education in Zhejiang and offering a model for similar studies in diverse educational settings.

### 6.1. Addressing Selection Bias and Ethical Considerations

In selecting teacher participants, schools were first stratified based on their geographical location and institutional type (private vs. state-run), ensuring an equitable distribution across the identified strata. Within each stratum, schools were randomly selected, and within those schools, teachers were invited to participate based on random sampling, ensuring each sub-group within the province’s educational ecosystem was fairly represented.

Ethical considerations were paramount throughout the research process. In compliance with ethical research standards, all participants were provided with detailed information about the study’s purpose, their role, and the confidentiality of their responses. Informed consent was obtained from every participant, emphasizing their right to withdraw at any time without consequence. The research protocol, including the sampling strategy and ethical safeguards, received approval from an institutional review board to ensure adherence to ethical guidelines and to mitigate potential biases.

Through these methodological and ethical rigors, the study endeavors to navigate the intricacies of assessing emotional competence and self-efficacy among preschool teachers in Zhejiang province. It aims not only to reflect the varied experiences of educators across different regional and institutional contexts but also to contribute valuable insights to the discourse on enhancing early-childhood education in China and beyond.

### 6.2. Measurement Items

In the study, the measurement of key constructs pertaining to preschool teachers’ performance draws on an array of scholarly work encompassing various aspects of teaching effectiveness and emotional intelligence. Creativity (CRE) is assessed through items influenced by Richards [91], Henriksen [92], and Glăveanu [93], emphasizing innovative thinking and creative problem-solving in educational contexts. Enthusiasm (ENT) is measured using scales derived from Keller et al. [94] and Bilz et al. [95], evaluating teachers’ passion and energy in engaging students. Organization (ORG) is gauged based on Emmer and Sabornie [96] and Hattie [97], focusing on teachers’ capability to manage classroom activities and foster a structured learning environment. Passion (PAS), as a key driver of effective teaching, is measured through items reflecting the insights of Day [98] and Palmer [99], highlighting the intensity and dedication of teachers to their profession. Patience (PAT), crucial for classroom management, is evaluated using scales from Spilt et al. [100] and Bakadorova et al. [101], focusing on teachers’ ability to maintain composure in varied teaching scenarios. Relationship management (RM), pivotal for positive teacher–student interactions, is assessed through scales from Jennings and Greenberg [102] and Pianta and Hamre [103], evaluating skills in managing and nurturing relationships with students. Social awareness (SA) is measured via items based on Jennings and Greenberg [102] and Downer et al. [104], focusing on teachers’ ability to perceive and appropriately respond to social cues in the classroom. Self-efficacy (SE) is evaluated using criteria from Tschannen-Moran and McMaster [105] and Klassen and Tze [67], assessing teachers’ confidence in their teaching capabilities and strategies. Self-awareness (SLA) is assessed through scales from Schonert-Reichl [106] and Brackett et al. [107], focusing on teachers’ understanding of their emotional states and strengths. Self-management (SM) is gauged using criteria from Brackett and Katulak [107] and Schonert-Reichl and Lawlor [106], evaluating teachers’ ability to regulate their emotions and behaviors effectively. Finally, teaching experience (TE) is evaluated through items inspired by Berliner [108] and Guo et al. [109], capturing the influence of years of teaching experience on teachers’ performance. These measurement items (refer to Appendix A) collectively offer a comprehensive assessment of the essential factors influencing preschool teachers’ performance, integrating aspects of emotional intelligence with practical teaching skills and experiences, thereby ensuring a thorough understanding of the dynamics at play in early-childhood education settings.

### 6.3. Data Analysis Techniques

In the current study, we will employ a sophisticated data analysis approach that integrates SmartPLS 4 for Partial Least Squares Structural Equation Modeling (PLS-SEM) and Necessary Condition Analysis (NCA), in line with recent methodological advancements in the field [110,111]. This dual-method strategy is specifically chosen to effectively dissect the intricate relationships between constructs such as self-efficacy, emotional competence, and preschool teachers’ performance, as well as to identify essential conditions within the dataset.

The model specification and analysis in SmartPLS 4 follows the guidelines of Hair et al. [110], who emphasize the utility of PLS-SEM in handling complex models with multiple latent variables and higher-order constructs. This is particularly relevant for multidimensional constructs like emotional competence, enabling an analysis of both individual dimensions and their collective impact, as suggested by Ringle et al. [111]. The path relationships proposed in the research hypotheses have been evaluated through path modeling, with the estimation of path coefficients and significance testing supported by bootstrapping (with 10,000 subsamples, two-tailed testing, at 0.05 significance level, fixed-seed random number generator) a technique recommended by Henseler et al. [112] for its robustness in PLS-SEM analysis.

Complementing PLS-SEM, NCA will be utilized as per the methodology outlined by Dul [113], to explore necessary conditions within the data. This analysis will follow the process described by Dul et al. (2020) [113], utilizing the same dataset as the SEM to uncover critical prerequisites for achieving desired outcomes, particularly in early-childhood educational contexts.

The integration of PLS-SEM and NCA in this study follows the emerging trend of combining different analytical methods to gain comprehensive insights into research data, as advocated by Sarstedt et al. [114].

## 7. Results

In analyzing the demographics of 580 preschool teachers in Zhejiang province (Table 1), key characteristics emerge that are crucial for interpreting this study’s outcomes. The age distribution shows a concentration in the middle-age brackets, with 37.9% of respondents aged 35–44 and 30.2% aged 25–34. This suggests a workforce that combines experience with potentially current pedagogical training. The gender disparity is notable, with a substantial 94.8% of the participants being female, reflecting global trends in early-childhood education and emphasizing the need to understand how this gender predominance might influence teaching styles and effectiveness. Educational qualifications of the respondents indicate a well-educated workforce: 51.7% hold a bachelor’s degree, and 34.5% have attained a master’s degree. This high level of formal education suggests familiarity with advanced educational theories and methodologies. However, the relatively small proportion (3.4%) of doctorate holders points to potential areas for further professional growth.

Experience levels vary, with 31.0% having 1–5 years and 25.9% having 6–10 years of teaching experience. This mix of newer and more experienced teachers adds diversity to the study, providing insights into how experience influences teaching practices.

Most respondents are classroom teachers (60.3%), followed by lead teachers/head teachers (20.7%), highlighting that the majority are directly involved in daily teaching activities. This distribution is essential for understanding the practical implications of the study’s findings in the context of early-childhood education. In summary, the demographics (Table 1) of the teachers in this study present a predominantly middle-aged, female, and well-educated group, with a blend of teaching experiences. These factors are key to understanding the dynamics of self-efficacy, emotional competence, and their impact on teacher performance in early-childhood education.

### 7.1. Measurement Model Assessment

**Figure 2:**
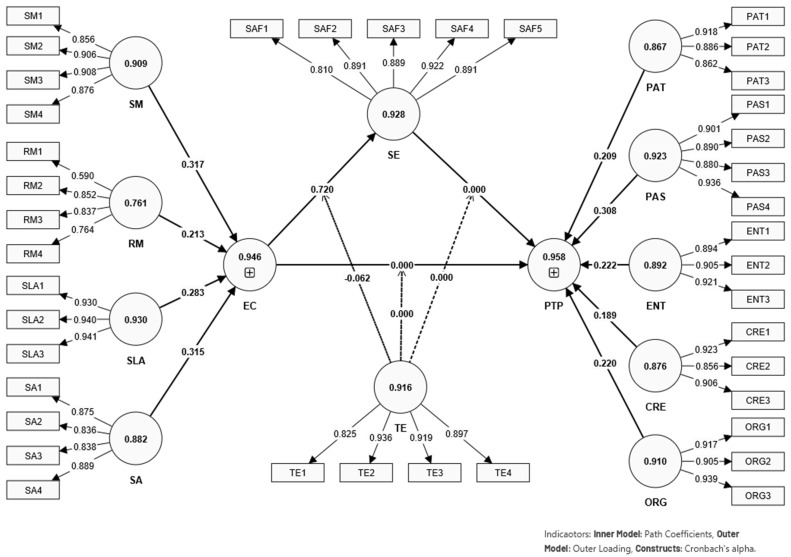
Illustration of measurement model (lower order construct).

The measurement model visualized in Figure 2 and expounded upon in Table 2 indicates a robust configuration of lower order constructs that contribute to the understanding of preschool teachers’ performance. Interpreting the detailed statistical results from Table 2, Table 3, Table 4 and Table 5 requires a close examination of the measurement models, validity assessments, and the higher-order construct loadings. These interpretations will be aligned with established methodological references in structural equation modeling (SEM) and statistical analysis.

Table 2 outlines the reliability and validity of the constructs within the measurement model. The outer loading (OL) values in the table represent the strength of the relationship between each item (e.g., CRE1, ENT1, ORG1, etc.) and its underlying construct (e.g., creativity, enthusiasm, organization, etc.). High OL values, which are all above the commonly accepted threshold of 0.7 [115], indicate that each item strongly reflects its respective construct, suggesting good indicator reliability and construct validity within the measurement model except for the relationship management item RM1. Constructs like creativity (CRE), enthusiasm (ENT), and organization (ORG) demonstrate high composite reliability (CR) and Cronbach’s Alpha (CA), surpassing the acceptable threshold of 0.7, as recommended by Hair et al. [112], indicating internal consistency. The average variance extracted (AVE) for each construct exceeds the recommended value of 0.5, signifying satisfactory convergent validity [116]. The variance inflation factor (VIF) values are below the threshold of five, suggesting no multicollinearity issues.

The HTMT (Heterotrait-Monotrait of correlations is a statistical technique for assessing discriminant validity in business management research [117]) ratio of criterion presented in Table 3 is a relatively novel approach to assessing discriminant validity. All HTMT values are below the conservative threshold of 0.85, as proposed by Henseler et al. [118], confirming that the constructs are empirically distinct. This is crucial in SEM to ensure that each construct uniquely contributes to the model without significant overlap with others.

Table 4 utilizes the Fornell-Larcker criterion to assess discriminant validity, where the square root of each construct’s AVE is compared against its correlations with other constructs. The diagonal values, representing the square root of the AVEs, are larger than the off-diagonal values in their respective rows and columns, satisfying the criterion set forth by Fornell and Larcker [116] and affirming discriminant validity.

The higher-order construct loadings in Table 5 reveal the strength of the relationship between constructs like creativity, enthusiasm, and organization with the outcome variables of preschool teachers’ performance (PTP) and emotional competence (EC). The outer loadings are all substantial and statistically significant (*p* < 0.001), as indicated by the T statistics, which are well above the threshold of 1.96 for significance at the 0.05 level [119]. This suggests that these constructs are critical components of the higher-order construct and are influential in the model.

The statistical analysis presented across these tables confirms the reliability and validity of the constructs used in the study and supports the structural relationships posited in the research model. The high levels of reliability, convergent validity, and discriminant validity, along with the significant loadings of the higher-order constructs, underscore the robustness of the model in explaining the factors influencing preschool teachers’ performance. The results provide a strong foundation for further discussion and implications within the scope of the study.

### 7.2. Structural Model Assessment

**Figure 3:**
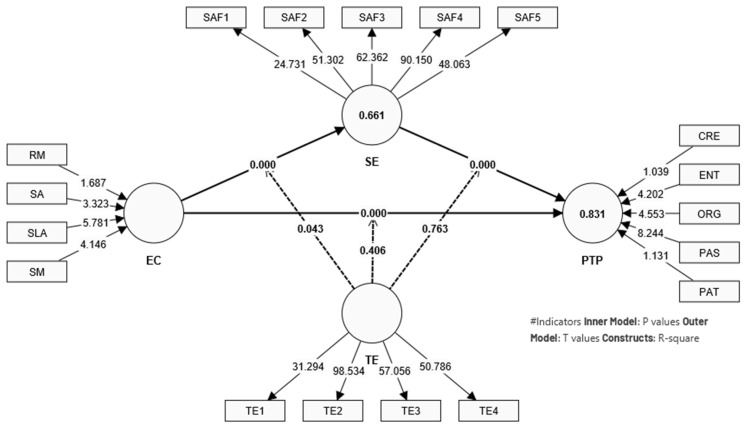
Illustration of structural model (higher order construct).

**Figure 4:**
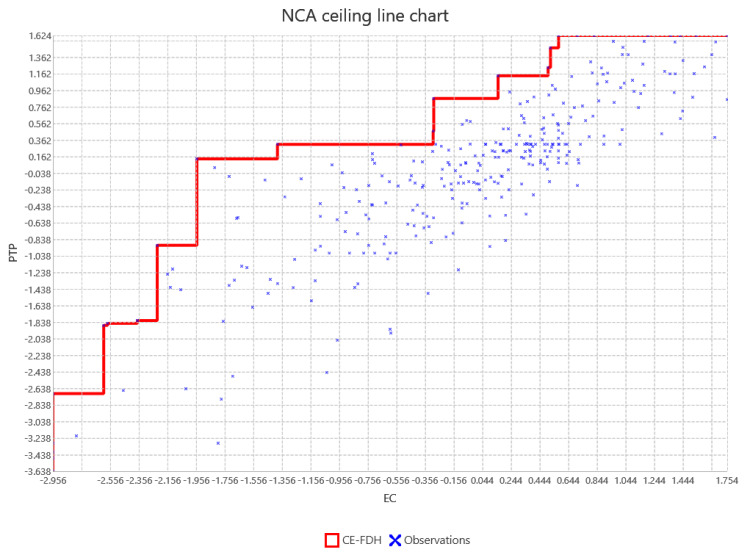
Scatter plot of outliers: emotional competence–preschool teachers’ performance (for interpretation of the references to color in this figure legend).

The structural model visualized in Figure 3 and scatter plot of outliers for NCA with Figure 4 and expounded upon in Table 6, Table 7, Table 8 and Table 9 which indicates a robust configuration of higher order constructs that contribute to the understanding of preschool teachers’ performance. Table 6 presents the statistics of the structural model, evaluating the mediation and moderation effects within the context of preschool teacher performance.

The results support hypotheses H1, H2, H3, and H6, indicating that self-efficacy (SE) positively predicts preschool teachers’ performance (PTP), emotional competence (EC) positively predicts self-efficacy, and directly impacts PTP. Notably, self-efficacy also mediates the relationship between EC and PTP, while teaching experience (TE) moderates the relationship between EC and SE. However, the moderating effect of TE on the relationship between EC and PTP, and between SE and PTP, was not supported, as indicated by the non-significant *p*-values. These findings align with the theoretical expectations posited by Bandura’s Theory of Self-Efficacy and emphasize the central role of emotional competence in teaching performance (Figure 2).

Table 7, detailing the model fit statistics, shows high R^2^ values for both PTP and SE, which signify that the model explains a substantial proportion of the variance in these constructs. The R^2^ adjusted and Q^2^ (predictive relevance, measures whether a model has predictive relevance or not [120]) predict values further affirm the model’s predictive accuracy and the model fit, indicating robustness and relevance for the educational context.

The analysis presented in Table 8 elucidates the role of emotional competence (EC) as not just a contributory factor but a necessary condition for preschool teachers’ performance (PTP), as underscored by Dul (2016) in the conceptualization of Necessary Condition Analysis (NCA). The table reveals an original effect size of 0.256 for EC, with a significant permutation *p*-value [113], indicating that a threshold level of EC is imperative for achieving any given level of PTP. Conversely, self-efficacy (SE) does not emerge as a necessary condition, signifying that while SE contributes to PTP, its absence does not preclude high performance.

In Table 9, the “Bottleneck table for emotional competence–preschool teachers’ performance”, a gradient of necessity for EC is exhibited, with the requirement of EC intensifying at higher performance percentiles. This gradient aligns with the principles of the CE-FDH approach, indicating that to reach the zenith of PTP, a substantial presence of EC is indispensable [113]. This finding is pivotal for educational interventions, highlighting that elevating EC could unlock higher levels of teacher performance.

Table 10, “Summary of findings for preschool teachers’ performance”, encapsulates the overarching insights from the structural model and NCA results. The table reinforces EC as a significant determinant and necessary condition for PTP, aligning with findings from Table 8 and Table 9. The significant but not relevant necessary condition results for SE and TE indicate that, while these factors are influential in determining PTP, they may not be as critical in terms of being limiting factors. This distinction between being a significant determinant and a necessary condition is crucial, as it suggests that improving SE and TE could enhance PTP, but their absence may not be as detrimental as the absence of EC.

The statistical analysis demonstrates that emotional competence is a critical factor in enhancing preschool teachers’ performance, with self-efficacy acting as an important intermediary in this process. This study’s innovative integration of PLS-SEM and NCA offers a comprehensive understanding of the factors contributing to effective teaching, providing empirical support for the integration of Bandura’s, Goleman’s, and Bronfenbrenner’s theories in the context of preschool education.

## 8. Discussion

The findings from the structural model and the Necessary Condition Analysis (NCA) concerning preschool teachers’ performance are enriched by the integration of the extant literature on self-efficacy, emotional competence, and teaching performance. Cocca [61] and Guo [62] reinforce the identified pathway from self-efficacy to teaching performance, with Guo emphasizing the role of collaboration and children’s engagement as key factors that bolster this relationship. This complements the study’s findings where self-efficacy is a significant predictor of performance (H1).

Garner [60] and Fu [81] discuss how emotional competence, encompassing emotion knowledge and emotional labor, significantly impacts preschool teachers’ performance. These findings align with our study’s results, where self-efficacy mediated the relationship between emotional competence and performance (H4), with a path coefficient of 0.250 (*p* < 0.000). The mediating role of self-efficacy is reinforced by the works of Valente [54], Kikas [82], and Şahin [49], who highlight how emotional intelligence and support boost self-efficacy, thus enhancing teaching performance. Pakarinen [77] furthers this discussion by emphasizing the quality of emotional support in fostering social competence, supporting our study’s finding that emotional competence directly influences preschool teachers’ performance (H3) with a substantial effect size (f^2^ = 0.656).

The literature presents mixed findings on the role of teaching experience in influencing the relationship between emotional competence and performance. Studies by Brown [83] and Valente [54] suggest that emotional intelligence significantly impacts teacher performance, which our study supports, albeit finding no significant moderation effect of teaching experience on performance (H5). In contrast, Bailey [84] and Garner [60] emphasize the importance of emotional support in the classroom, which could imply that with greater experience, teachers might better leverage emotional competence to enhance performance. However, our study’s analysis indicated that teaching experience did not significantly moderate the relationship between self-efficacy and performance (H6), presenting an area for further investigation.

While Bay [63] and Cocca [61] report a positive correlation between self-efficacy and performance, Guo [62] and Infurna [66] offer contrasting views, with some evidence suggesting that teaching experience does not impact self-efficacy. This mirrors the complexity identified in our study, where teaching experience was found to moderate the relationship between emotional competence and self-efficacy (H7), with a path coefficient of −0.060 (*p* = 0.043), yet it did not significantly moderate the relationship between self-efficacy and performance.

The consensus in the literature on the positive relationship between emotional competence and self-efficacy is consistent with our findings. Penrose [87], Wu [88], and Valente [54] outline this relationship, which is often mediated by teaching performance and work engagement, reinforcing the significance of emotional competence as a predictor of self-efficacy (H2) with a path coefficient of 0.731 (*p* < 0.000) and a large effect size (f^2^ = 1.263).

The literature provides multifaceted perspectives on the role of self-efficacy and emotional competence in influencing teaching performance, with an emphasis on the complexity of these relationships and the varying influence of teaching experience. Our study contributes to this discourse by offering empirical evidence of these dynamics within the context of preschool education, highlighting significant pathways and conditions that underpin effective teaching. The need for further research is evident to clarify the role of teaching experience in these relationships and to explore the implications for teacher training and development programs.

### 8.1. Implications of This Study

#### 8.1.1. Theoretical Implications

The study’s exploration into the factors influencing preschool teachers’ performance provides empirical substantiation to Bandura’s Theory of Self-Efficacy, Goleman’s Emotional Intelligence Theory, and Bronfenbrenner’s Ecological Systems Theory, offering significant theoretical implications for the field of early-childhood education.

Consistent with Bandura’s [47] seminal work, the study affirms self-efficacy as a critical predictor of teaching performance. It elucidates that teachers who believe in their instructional abilities are more adept at executing effective teaching strategies, a finding that echoes Bandura’s assertion of self-efficacy’s transformative potential. Moreover, the mediating role of self-efficacy between emotional competence and teaching performance is evident, suggesting that self-efficacy is not merely a personal trait but an actionable mediator that translates emotional competence into enhanced performance.

Reflecting on Goleman’s [25] constructs of emotional intelligence, the study reveals that emotional competence is both a significant predictor and a necessary condition for effective teaching. This dual role emphasizes the criticality of emotional intelligence in the educational sphere, highlighting the necessity for teachers to possess the skills to understand and manage their emotions alongside those of their students. The findings suggest that interventions aimed at bolstering teachers’ emotional competence could result in marked improvements in teaching performance.

Through the lens of Bronfenbrenner’s [26] theory, the study situates the moderating effect of teaching experience within the broader ecological systems affecting individual development. It illustrates that the work environment and related social support systems play a pivotal role in shaping teachers’ self-efficacy and emotional competence. This underscores the importance of systemic factors and supports the notion that organizational and policy interventions are crucial for creating environments conducive to the development of key teacher attributes.

In sum, this study not only corroborates but also extends these foundational theories by demonstrating the complex interplay between emotional competence, self-efficacy, and the ecological context in determining preschool teachers’ performance. The findings advocate for a holistic approach to teacher development, one that encompasses both the enhancement of individual teacher capacities and the cultivation of supportive educational ecosystems. Such a multifaceted strategy promises to elevate the caliber of early-childhood education, aligning with the aspirational goals of educational excellence and teacher empowerment.

#### 8.1.2. Practical Implications

This research, delving into the nexus of emotional competence, self-efficacy, and preschool teachers’ performance within the unique educational landscape of China, underscores profound practical implications, particularly in the context of a nation under a communist regime where educational policies and practices are centrally managed. The study’s insights highlight the necessity of embedding emotional intelligence skills within teacher training and professional development frameworks. Beyond traditional pedagogical skills, these programs must prioritize fostering empathy, self-regulation, and communication, aligning with the holistic educational objectives promoted by China’s educational reforms.

The imperative to enhance teachers’ self-efficacy resonates strongly within China’s educational milieu, where the central government’s directives seek to elevate the teaching quality across the board. Strategies such as mentorship, reflective practices, and narratives of success become even more critical in reinforcing teachers’ confidence in their abilities, resonating with the nationwide push towards educational excellence.

Curricular reforms and classroom practices must also reflect an integrated approach to emotional learning, a move that aligns with China’s broader educational goals of fostering well-rounded, socially competent students. Teachers modeling emotional competence becomes a cornerstone in this endeavor, ensuring that classroom dynamics are effectively managed and a nurturing learning environment is cultivated.

Policy and organizational shifts within educational institutions are paramount. Acknowledging the emotional labor of teaching, especially in a system that values collective success, necessitates supportive work environments, access to mental health resources, and policies that acknowledge the comprehensive nature of teaching. These changes are crucial in realizing the Chinese educational sector’s ambition of not only academic excellence but also the well-being of its educators.

Moreover, the study emphasizes the importance of regular assessments of teachers’ emotional competence and self-efficacy, tailored professional development, and a continuous feedback mechanism. Such measures are essential for aligning teaching practices with the evolving educational standards and expectations of China’s centrally managed educational system.

The call for collaborative practices and community engagement speaks to the communal ethos of Chinese society, underscoring the potential for collective efforts to enhance educational outcomes. This collaborative spirit extends to the necessity for ongoing research into the domains of emotional competence and self-efficacy in teaching, urging the establishment of professional learning communities that are in sync with the latest educational strategies and research, thereby contributing to the sustenance and enhancement of early-childhood-education quality.

In sum, this study advocates for a comprehensive strategy that not only fosters individual teacher development in crucial competencies but also aligns with China’s systemic educational support mechanisms. Such an approach is pivotal for elevating teaching standards, fostering effective learning environments, and ultimately enriching the educational journey of young learners in their most formative years, reflecting the unique characteristics and aspirations of China’s education system under its governance model.

### 8.2. Conclusion and Future Directions

This study, situated at the intersection of emotional competence, self-efficacy, and preschool teachers’ performance, has revealed critical insights with significant implications for early-childhood education. By empirically validating the interrelatedness of these constructs, it has shed light on the essential role emotional competence plays in enhancing teaching performance, mediated by the pivotal element of self-efficacy. These findings resonate with the foundational principles laid out in Bandura’s Theory of Self-Efficacy, Goleman’s Emotional Intelligence Theory, and Bronfenbrenner’s Ecological Systems Theory, thereby enriching the existing educational literature.

The study has underscored the necessity for educational systems to prioritize the development of emotional competence in preschool teachers, recognizing it, not only as a skill, but as a fundamental component of effective teaching. The emphasis on self-efficacy as a mediator provides a blueprint for structuring teacher training and development programs, highlighting the need for a supportive environment that fosters confidence and competence in teachers. Furthermore, the findings of this study advocate for a broader, systemic approach to teacher development, one that transcends traditional pedagogical training and delves into the psychological and emotional aspects of teaching.

In terms of future research, several avenues present themselves. First, there is a need for longitudinal studies to track the long-term impact of enhanced emotional competence and self-efficacy on teaching performance and student outcomes. This would provide a more comprehensive understanding of the temporal dynamics of these relationships.

Another direction would be to replicate this study in different cultural and educational contexts. Since cultural norms and educational systems vary widely, it would be insightful to explore whether the relationships among emotional competence, self-efficacy, and performance hold true across different settings or if there are context-specific variations.

Future research should also explore the role of additional moderating variables, such as teachers’ stress levels, work–life balance, and institutional support, in the relationship between emotional competence, self-efficacy, and teaching performance. This would add depth to the current understanding of the factors that influence teacher effectiveness.

Moreover, studies focusing on the development and testing of specific interventions or training programs designed to enhance emotional competence and self-efficacy in preschool teachers would be invaluable. The effectiveness of such programs in real-world educational settings could provide practical insights for educators and policymakers.

Finally, there is a scope for incorporating technological advancements, such as artificial intelligence and machine learning, to assess and enhance emotional competence and self-efficacy among teachers. This could open up innovative methods for teacher training and development.

In summary, the study paves the way for a reimagined approach to teacher development, one that is holistic, emotionally intelligent, and efficacy-oriented. The path ahead is ripe with opportunities for further exploration and innovation in the field of early-childhood education, promising to unlock new frontiers in teaching effectiveness and educational excellence.

## Figures and Tables

**Table 1:** Respondents’ demographics.

Demographics	Variables	Number (Count, Percentage)
Age	Under 25	30 (5.2%)
	25–34	175 (30.2%)
	35–44	220 (37.9%)
	45–54	110 (19.0%)
	55–64	40 (6.9%)
	65 or above	5 (0.9%)
Gender	Female	550 (94.8%)
	Male	30 (5.2%)
Educational Qualification	High school diploma or equivalent	20 (3.4%)
	Associate degree	40 (6.9%)
	Bachelor’s degree	300 (51.7%)
	Master’s degree	200 (34.5%)
	Doctorate or higher	20 (3.4%)
Years of Teaching Experience	Less than 1 year	50 (8.6%)
	1–5 years	180 (31.0%)
	6–10 years	150 (25.9%)
	11–15 years	100 (17.2%)
	16–20 years	60 (10.3%)
	More than 20 years	40 (6.9%)
Current Position	Classroom teacher	350 (60.3%)
	Lead teacher/Head teacher	120 (20.7%)
	Teacher’s assistant/aide	50 (8.6%)
	Administrative role	40 (6.9%)
	Other	20 (3.4%)

**Table 2:** Measurement model statistics (lower order construct).

Construct	CODE	OL	VIF	CA	CR (rho_a)	AVE
CRE	CRE1	0.923	2.984	0.876	0.881	0.802
	CRE2	0.856	1.973			
	CRE3	0.906	2.758			
ENT	ENT1	0.894	2.342	0.892	0.892	0.822
	ENT2	0.905	2.755			
	ENT3	0.921	3.083			
ORG	ORG1	0.917	3.124	0.910	0.910	0.847
	ORG2	0.905	2.690			
	ORG3	0.939	3.749			
PAS	PAS1	0.901	3.171	0.923	0.924	0.814
	PAS2	0.890	2.918			
	PAS3	0.880	2.931			
	PAS4	0.936	4.522			
PAT	PAT1	0.918	2.816	0.867	0.869	0.790
	PAT2	0.886	2.274			
	PAT3	0.862	2.063			
RM	RM1	0.590	1.178	0.761	0.785	0.590
	RM2	0.852	1.919			
	RM3	0.837	1.921			
	RM4	0.764	1.518			
SA	SA1	0.875	2.519	0.882	0.885	0.739
	SA2	0.836	2.010			
	SA3	0.838	2.108			
	SA4	0.889	2.773			
SE	SAF1	0.810	2.256	0.928	0.929	0.777
	SAF2	0.891	3.795			
	SAF3	0.889	3.431			
	SAF4	0.922	4.572			
	SAF5	0.891	3.296			
SLA	SLA1	0.930	3.516	0.930	0.931	0.878
	SLA2	0.940	3.900			
	SLA3	0.941	3.953			
SM	SM1	0.856	2.322	0.909	0.911	0.786
	SM2	0.906	3.251			
	SM3	0.908	3.377			
	SM4	0.876	2.580			
TE	TE1	0.825	1.967	0.916	0.918	0.801
	TE2	0.936	4.662			
	TE3	0.919	4.020			
	TE4	0.897	3.318			

CRE-> creativity, ENT-> enthusiasm, ORG-> organization, PAS-> passion, PAT-> patience, RM-> relationship management, SA-> social awareness, SE-> self-efficacy, SLA-> self-awareness, SM-> self-management, TE-> teaching experience, OL-> outer loading, VIF-> variance inflation factor, CA-> Cronbach’s Alpha, CR (rho_a)-> composite reliability, AVE-> average variance extracted.

**Table 3:** Discriminant validity (HTMT).

	CRE	ENT	ORG	PAS	PAT	RM	SA	SE	SLA	SM	TE	TE × EC	TE × SE
CRE													
ENT	0.634												
ORG	0.620	0.820											
PAS	0.689	0.806	0.815										
PAT	0.812	0.783	0.710	0.849									
RM	0.547	0.739	0.763	0.782	0.627								
SA	0.607	0.824	0.795	0.849	0.699	0.829							
SE	0.640	0.799	0.800	0.873	0.797	0.714	0.825						
SLA	0.632	0.815	0.766	0.868	0.698	0.839	0.834	0.814	-				
SM	0.604	0.815	0.774	0.779	0.715	0.693	0.827	0.740	0.760	-			
TE	0.313	0.425	0.399	0.428	0.415	0.308	0.393	0.439	0.395	0.339	-		
TE × EC	0.222	0.331	0.301	0.340	0.357	0.279	0.301	0.353	0.279	0.303	0.174	-	
TE × SE	0.130	0.380	0.379	0.370	0.374	0.278	0.349	0.481	0.272	0.326	0.216	0.847	-

CRE-> creativity, ENT-> enthusiasm, ORG-> organization, PAS-> passion, PAT-> patience, RM-> relationship management, SA-> social awareness, SE-> self-efficacy, SLA-> self-awareness, SM-> self-management, TE-> teaching experience, SE-> self-efficacy, EC-> emotional competence.

**Table 4:** Discriminant validity (Fornell-Larcker criterion).

Variables	CRE	ENT	ORG	PAS	PAT	RM	SA	SE	SLA	SM	TE
CRE	0.895										
ENT	0.562	0.907									
ORG	0.554	0.739	0.920								
PAS	0.621	0.822	0.747	0.902							
PAT	0.709	0.690	0.632	0.760	0.889						
RM	0.446	0.612	0.635	0.660	0.507	0.768					
SA	0.533	0.757	0.714	0.767	0.613	0.683	0.860				
SE	0.578	0.728	0.736	0.808	0.715	0.601	0.748	0.881			
SLA	0.572	0.743	0.704	0.805	0.628	0.711	0.816	0.758	0.937		
SM	0.541	0.735	0.705	0.715	0.636	0.577	0.743	0.681	0.700	0.887	
TE	0.281	0.384	0.364	0.393	0.370	0.266	0.353	0.404	0.365	0.310	0.895

CRE-> creativity, ENT-> enthusiasm, ORG-> organization, PAS-> passion, PAT-> patience, RM-> relationship management, SA-> social awareness, SE-> self-efficacy, SLA-> self-awareness, SM-> self-management, TE-> teaching experience.

**Table 5:** Higher order model (outer loading).

Hypothesis Path	Original Sample (O)	Sample Mean (M)	Standard Deviation (STDEV)	T Statistics (|O/STDEV|)	*p* Values
CRE-> PTP	0.684	0.682	0.061	11.122	0.000
ENT-> PTP	0.903	0.900	0.019	47.898	0.000
ORG-> PTP	0.880	0.877	0.021	42.150	0.000
PAS-> PTP	0.957	0.954	0.012	77.494	0.000
PAT-> PTP	0.804	0.801	0.044	18.113	0.000
RM-> EC	0.767	0.764	0.031	25.036	0.000
SA-> EC	0.924	0.921	0.022	41.667	0.000
SLA-> EC	0.939	0.936	0.017	56.589	0.000
SM-> EC	0.868	0.865	0.034	25.232	0.000

CRE-> creativity, ENT-> enthusiasm, ORG-> organization, PAS-> passion, PAT-> patience, RM-> relationship management, SA-> social awareness, SLA-> self-awareness, SM-> self-management, PTP-> preschool teachers’ performance, EC-> emotional competence.

**Table 6:** Statistics of structural model (mediation and moderation effect).

Hypo	PATH	Original Sample (O)	Standard Deviation (STDEV)	T Statistics (|O/STDEV|)	*p* Values	f^2^	Support
H1	SE -> PTP	0.342	0.061	5.601	0.000	0.196	Yes
H2	EC -> SE	0.731	0.034	21.657	0.000	1.263	Yes
H3	EC -> PTP	0.578	0.058	9.903	0.000	0.656	Yes
H4	EC -> SE -> PTP	0.250	0.046	5.405	0.000		Yes
H5	TE × EC -> PTP	−0.035	0.042	0.830	0.406	0.004	No
H6	TE × SE -> PTP	0.012	0.040	0.302	0.763	0.000	No
H7	TE × EC -> SE	−0.060	0.029	2.025	0.043	0.022	Yes

SE-> self-efficacy, TE-> teaching experience, PTP-> preschool teachers’ performance, EC-> emotional competence.

**Table 7:** Model fit statistics.

Dependent Variables	R^2^	R^2^ Adjusted	Q^2^ Predict	RMSE	MAE
PTP	0.831	0.829	0.773	0.480	0.355
SE	0.661	0.658	0.642	0.603	0.425

SE-> self-efficacy, PTP-> preschool teachers’ performance.

**Table 8:** NCA effect size (CE-FDH).

Constructs	Original Effect Size	95.00%	Permutation *p* Value
EC	0.256	0.049	0.000
SE	0.000	0.014	0.806
TE	0.000	0.048	1.000

TE-> teaching experience, EC-> emotional competence, SE-> self-efficacy.

**Table 9:** Bottleneck table for emotional competence–preschool teachers’ performance (CE-FDH).

Percentages	PTP	EC	SE	TE
0.00%	−3.638	0.000	0.000	0.000
10.00%	−3.112	0.000	0.000	0.000
20.00%	−2.586	1.829	0.000	0.000
30.00%	−2.059	1.829	0.000	0.000
40.00%	−1.533	3.049	0.000	0.000
50.00%	−1.007	3.049	0.000	0.000
60.00%	−0.481	4.878	0.000	0.000
70.00%	0.045	4.878	0.000	0.000
80.00%	0.571	32.622	0.000	0.000
90.00%	1.098	49.085	0.000	0.000
100.00%	1.624	71.341	0.000	0.000

TE-> teaching experience, EC-> emotional competence, SE-> self-efficacy, PTP-> preschool teachers’ performance.

**Table 10:** Summary of findings for preschool teachers’ performance.

Construct	PLS-SEM Results	NCA results
EC	Significant determinant	Significant and relevant necessary condition
SE	Significant determinant	Significant but not relevant necessary condition
TE	Significant determinant	Significant but not relevant necessary condition

TE-> teaching experience, EC-> emotional competence, SE-> self-efficacy.

## Data Availability

Data will be made available with reasonable request to the corresponding author.

## Appendix A

**Construct**	**Code**	**ITEMS**	**Sources**
CRE	CRE1	I often come up with new and fun ways to teach my students.	[81,82,83]
	CRE2	I enjoy using arts and crafts to enhance learning experiences.	
	CRE3	I regularly incorporate play and imaginative activities into my teaching.	
ENT	ENT1	I feel excited about teaching every day.	[84,85]
	ENT2	I show a lot of energy and enthusiasm in my classroom.	
	ENT3	I am passionate about making learning enjoyable for my students.	
ORG	ORG1	I plan my lessons well in advance and stick to my schedule.	[86,87]
	ORG2	My classroom is well-organized and conducive to learning.	
	ORG3	I keep track of my students’ progress and needs systematically.	
PAS	PAS1	I deeply care about the educational and personal growth of my students.	[88,89]
	PAS2	Teaching is more than a job to me; it’s a passion.	
	PAS3	I am committed to continuous learning and improvement as a teacher.	
	PAS4	I go above and beyond to ensure the success of my students.	
PAT	PAT1	I remain calm and patient when students struggle with learning.	[90,91]
	PAT2	I take time to understand and address each student’s individual needs.	
	PAT3	I handle classroom disruptions calmly and patiently.	
RM	RM1	I effectively resolve conflicts among students in my classroom.	[92,93]
	RM2	I build trusting and positive relationships with all my students.	
	RM3	I communicate effectively with parents about their child’s progress.	
	RM4	I work well with other teachers and staff to create a supportive learning environment.	
SA	SA1	I am aware of the social and emotional cues of my students.	
	SA2	I understand the diverse cultural backgrounds of my students.	[92,94]
	SA3	I adjust my teaching to accommodate the different learning styles in my classroom.	
	SA4	I recognize and respond appropriately to my students’ emotional needs.	
SE	SAF1	I am confident in my ability to teach effectively.	[56,95]
	SAF2	I believe I can handle challenging situations in my classroom.	
	SAF3	I feel capable of motivating my students to learn.	
	SAF4	I believe I can make a positive difference in my students’ learning.	
	SAF5	I am confident in my ability to manage classroom behavior.	
SLA	SLA1	I am aware of how my mood and behavior affect my students.	[96,97]
	SLA2	I understand my strengths and weaknesses as a teacher.	
	SLA3	I reflect on my teaching practices to improve them.	
SM	SM1	I manage stress effectively to maintain a positive classroom environment.	[96,97]
	SM2	I stay calm and composed even in challenging teaching situations.	
	SM3	I adapt my teaching strategies when faced with unexpected issues.	
	SM4	I maintain a balanced and healthy approach to work and personal life.	
TE	TE1	I have several years of experience in teaching preschool.	[98,99]
	TE2	I have experience working with diverse groups of preschoolers.	
	TE3	Over the years, I have developed a range of effective teaching strategies.	
	TE4	My teaching methods have evolved based on my experiences in the classroom.	
CRE-> creativity, ENT-> enthusiasm, ORG-> organization, PAS-> passion, PAT-> patience, RM-> relationship management, SA-> social awareness, SE-> self-efficacy, SLA-> self-awareness, SM-> self-management, TE-> teaching.			
